# Systems biology approach reveals a common molecular basis for COVID-19 and non-alcoholic fatty liver disease (NAFLD)

**DOI:** 10.1186/s40001-022-00865-y

**Published:** 2022-11-15

**Authors:** Shi-Tao Jiang, Yao-Ge Liu, Lei Zhang, Xin-Ting Sang, Yi-Yao Xu, Xin Lu

**Affiliations:** Department of Liver Surgery, State Key Laboratory of Complex Severe and Rare Diseases, Peking Union Medical College Hospital, Chinese Academy of Medical Sciences and Peking Union Medical College, Beijing, China

**Keywords:** COVID-19, Non-alcoholic fatty liver disease, Differentially expressed genes, Network analysis, Drug

## Abstract

**Background:**

Patients with non-alcoholic fatty liver disease (NAFLD) may be more susceptible to coronavirus disease 2019 (COVID-19) and even more likely to suffer from severe COVID-19. Whether there is a common molecular pathological basis for COVID-19 and NAFLD remains to be identified. The present study aimed to elucidate the transcriptional alterations shared by COVID-19 and NAFLD and to identify potential compounds targeting both diseases.

**Methods:**

Differentially expressed genes (DEGs) for COVID-19 and NAFLD were extracted from the GSE147507 and GSE89632 datasets, and common DEGs were identified using the Venn diagram. Subsequently, we constructed a protein–protein interaction (PPI) network based on the common DEGs and extracted hub genes. Then, we performed gene ontology (GO) and pathway analysis of common DEGs. In addition, transcription factors (TFs) and miRNAs regulatory networks were constructed, and drug candidates were identified.

**Results:**

We identified a total of 62 common DEGs for COVID-19 and NAFLD. The 10 hub genes extracted based on the PPI network were IL6, IL1B, PTGS2, JUN, FOS, ATF3, SOCS3, CSF3, NFKB2, and HBEGF. In addition, we also constructed TFs–DEGs, miRNAs–DEGs, and protein–drug interaction networks, demonstrating the complex regulatory relationships of common DEGs.

**Conclusion:**

We successfully extracted 10 hub genes that could be used as novel therapeutic targets for COVID-19 and NAFLD. In addition, based on common DEGs, we propose some potential drugs that may benefit patients with COVID-19 and NAFLD.

**Supplementary Information:**

The online version contains supplementary material available at 10.1186/s40001-022-00865-y.

## Introduction

Coronavirus disease 2019 (COVID-19) is an infectious disease caused by severe acute respiratory syndrome coronavirus 2 (SARS-CoV-2) [1]. Although most patients with COVID-19 present with acute, self-limiting episodes [2], the disease can be fatal. Approximately 3.6% of infected individuals will die from it [3]. Disease severity is significantly associated with impaired immune response and co-morbidities [4]. Pre-existing conditions, such as non-alcoholic fatty liver disease (NAFLD), play a crucial role in COVID-19 disease progression [5]. Studies have shown that COVID-19-related liver injury is more common in patients with NAFLD. Moreover, the patients are more likely to advance to cholestatic liver failure or secondary sclerosing cholangitis [6]. Given the continued prevalence of COVID-19 and the rapid increase in the prevalence of NAFLD worldwide [7, 8], researchers have begun to focus on how to help patients with NAFLD better cope with the challenges presented by COVID-19.

The hepatic manifestations of NAFLD range from simple steatosis (SS) to non-alcoholic steatohepatitis (NASH) [9]. Although NAFLD is primarily a metabolic disorder, it involves several immune cell-mediated inflammatory processes, especially when it reaches the NASH stage [10]. At this point, inflammation becomes an essential component of disease progression. Studies have shown that NAFLD patients are more susceptible to SARS-CoV2 infection, possibly because NAFLD patients have more expression of receptors on the cell surface that SARS-CoV-2 can bind to [11]. Recently Ji et al. found that NAFLD patients were more prone to severe COVID-19 when infected with SARS-CoV-2 than non-NAFLD patients. They speculated that this might be due to NAFLD patients' already existing immune dysfunction [12]. Therefore, to improve the treatment of COVID-19 patients with co-morbidities, we can start by understanding the molecular interactions shared by COVID-19 and NAFLD.

**Fig. 1:**
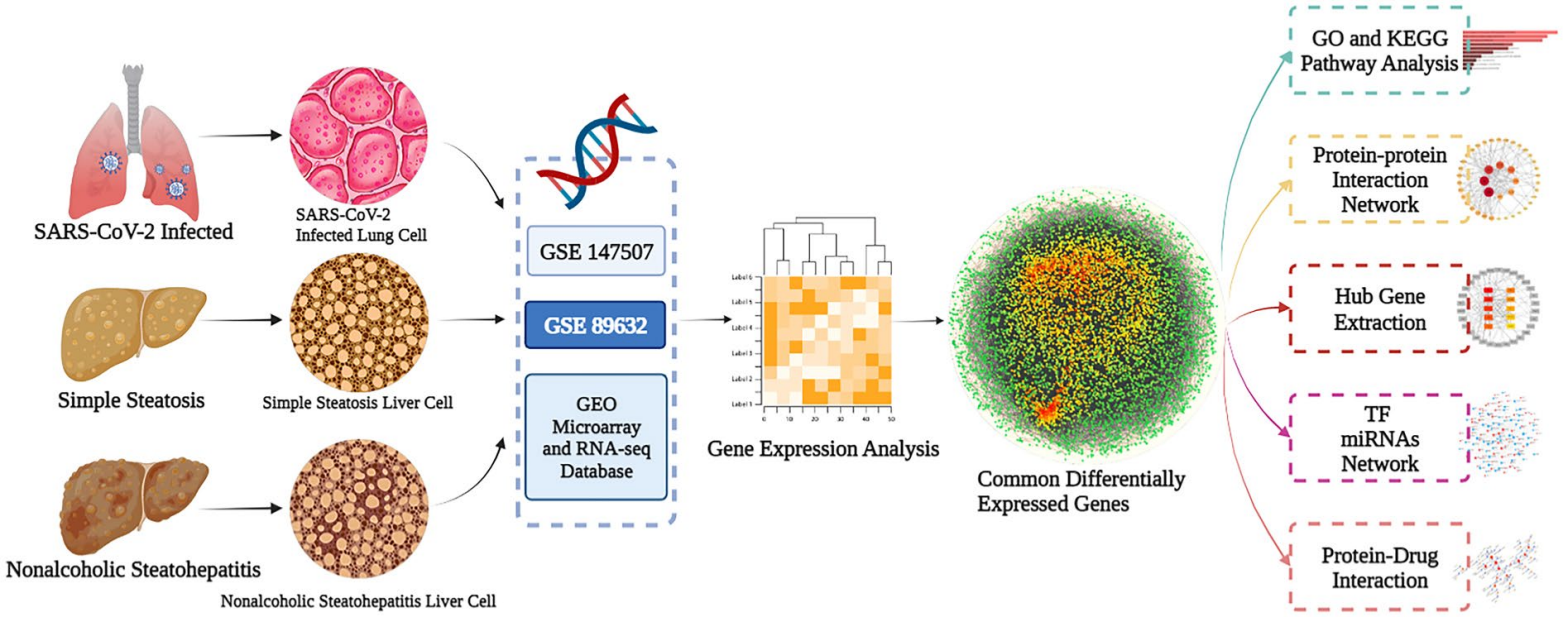
Study design and workflow diagram

In this investigation, we hypothesized that the two diseases shared similar transcriptomic alterations. By performing a systems biology analysis of the common DEGs of the two diseases, we elucidated the impact of the NAFLD environment on SARS-CoV-2 infected patients at the molecular scale. Further, we identified some potential compounds that could be used to treat COVID-19 and NAFLD. Figure 1 shows in detail the main procedures and methods of this study.

## Material and methods

### Basic information of the datasets

This study used two National Center for Biotechnology Information (NCBI) GEO datasets to explore common genetic correlations between COVID-19 and NAFLD. GSE147507 was the GEO accession number for the COVID-19 dataset [13]. This dataset included biologically independent triplicates of primary human lung epithelium (NHBE), transformed lung alveolar (A549) cells, and transformed lung-derived Calu-3 cells that were mock-treated or infected with SARS-CoV-2. In addition, uninfected human lung biopsies from a 72-year-old male and a 60-year-old female were used as biological replicates. Furthermore, technical replicates were performed on lung samples obtained from a 74-year-old COVID-19-infected patient. The researchers used GPL18573 Illumina NextSeq 500 for RNA sequence extraction. In addition, the dataset GSE89632 included 63 participants (20 SS, 19 NASH, and 24 healthy controls). In the study included in this dataset, NAFLD activity score (NAS) greater than or equal to 5 correlated with a diagnosis of NASH, and biopsies with scores of less than 3 were diagnosed as “SS”. These samples were sequenced using a microarray called GPL14951 Illumina HumanHT-12 WG-DASL V4.0 R2 expression beadchip [14]. The summarized information of the datasets is shown in Table 1.

**Table 1:** Relevant information of GEO database and analysis results of differential expression genes

Disease	Accession number	Platform	Total DEGs	Positively regulated DEGs	Negatively regulated DEGs
COVID-19	GSE147507	GPL18573	1057	841	216
SS	GSE89632	GPL14951	473	156	317
NASH	GSE89632	GPL14951	487	209	278

GEO, Gene Expression Omnibus; DEGs, differential expression genes; COVID-19, coronavirus disease-2019; SS, simple steatosis; NASH, non-alcoholic steatohepatitis

In this study, the datasets GSE147507 and GSE89632 were used to obtain the DEGs of COVID-19, SS, and NASH, respectively. The subsequent analysis is performed in the R programming language (version 4.0.2). For the analysis of the raw counts of GSE147507, we used the DESeq2 package (version 1.36.0). In addition, we used the limma package with Benjamini–Hochberg multiple-testing correction to detect DEGs of GSE89632. Genes with a false discovery rate (FDR) of less than 0.05 and |log2FC| greater than 1 were deemed differentially expressed. Venn diagrams were generated using the online tool jvenn to obtain the common DEGs of COVID-19, SS, and NASH.

### Analysis of gene ontology and pathway enrichment

Enrichr (https://maayanlab.cloud/Enrichr/) is a comprehensive web-based tool for gene set enrichment analysis, used to perform gene ontology and pathway analysis with a focus on the KEGG, Biocarta, Reactome, and Wikipathways databases [15]. *P*-values < 0.05 were statistically significant and used to rank enrichment results quantitatively.

### Protein–protein interaction (PPI) network analysis

A PPI network based on the common DEGs was generated in the STRING (version 11.5) with a confidence score threshold of 0.4. Default settings were used for the rest of the parameters. Proteins not connected to the interaction network were filtered out [16]. The resulting protein interaction network was visualized using Cytoscape (version 3.9.1) [17].

### Extraction and analysis of the hub genes

In PPI networks, hub genes are those nodes with high connectivity. Hub genes are involved in more interactions throughout the network and therefore may be more critical than low-connected genes [18]. CytoHubba provides a user-friendly interface to explore the critical nodes in a biological network [19]. Maximal Clique Centrality (MCC) is one of 11 techniques to study networks from different perspectives [20]. Using MCC embedded in Cytohubba, we identified the top 10 hub genes from the PPI network. Furthermore, we did receiver operating characteristic (ROC) analysis to validate the ability of hub genes to classify different disease statuses. The visualization of the ROC analysis is provided in Additional file 1: Figure S1.

### Construction of regulatory networks of transcription factors (TFs) and miRNAs

TFs were crucial proteins involved in the regulation of gene transcription. These proteins specifically bind to DNA to control the complex system of genome expression [21]. In order to identify the topologically plausible TFs that could bind to the common DEGs, we used the JASPAR database integrated into the NetworkAnalyst. JASPAR is an open-access database of curated, non-redundant transcription factor (TF)-binding profiles stored as position frequency matrices (PFMs) for TFs across multiple species in six taxonomic groups [22]. Since the first official release of JASPAR in 2004, the research community has embraced it as the leading open-access database of such matrix profiles for TF binding sites. In 2014, NetworkAnalyst was introduced as a robust web-based visual analytics platform for gene expression data profiling, meta-analysis, and systems-level interpretation [23]. Researchers released the most recent version of NetworkAnalyst in 2019. NetworkAnalyst 3.0 also integrated the miRTarbase database, the most famous comprehensive miRNA–target interaction database [24]. We then used the miRTarBase database to select all miRNAs targeting this study's common DEGs [25].

### Screening of potential therapeutic compounds

A critical aspect of this investigation was the prediction of protein–drug interactions (PDI) and identifying potential therapeutic molecules. To this end, we utilized the Drug Signatures Database (DSigDB) integrated into Enrichr to investigate drug molecules that could act on the common DEGs described in this study [26]. DSigDB was a widely acknowledged new gene set resource that linked drugs/compounds with their target genes for gene enrichment analysis (GSEA).

### Analyses of disease–gene associations

DisGeNET (http://www.disgenet.org) is a knowledge management platform that integrates and standardizes data on disease-associated genes and variants from multiple sources. More than 24,000 diseases and traits, 17,000 genes, and 117,000 genomic variants were included in the most recent version of DisGeNET. It demonstrated the expanding knowledge of human genetic diseases [27]. Therefore, we further investigated gene and disease associations using DisGeNET, which had been integrated into NetworkAnalyst, to identify common DEGs associated with diseases and chronic problems.

## Results

### Identification of DEGs and common DEGs among COVID-19, SS, and NASH

**Fig. 2:**
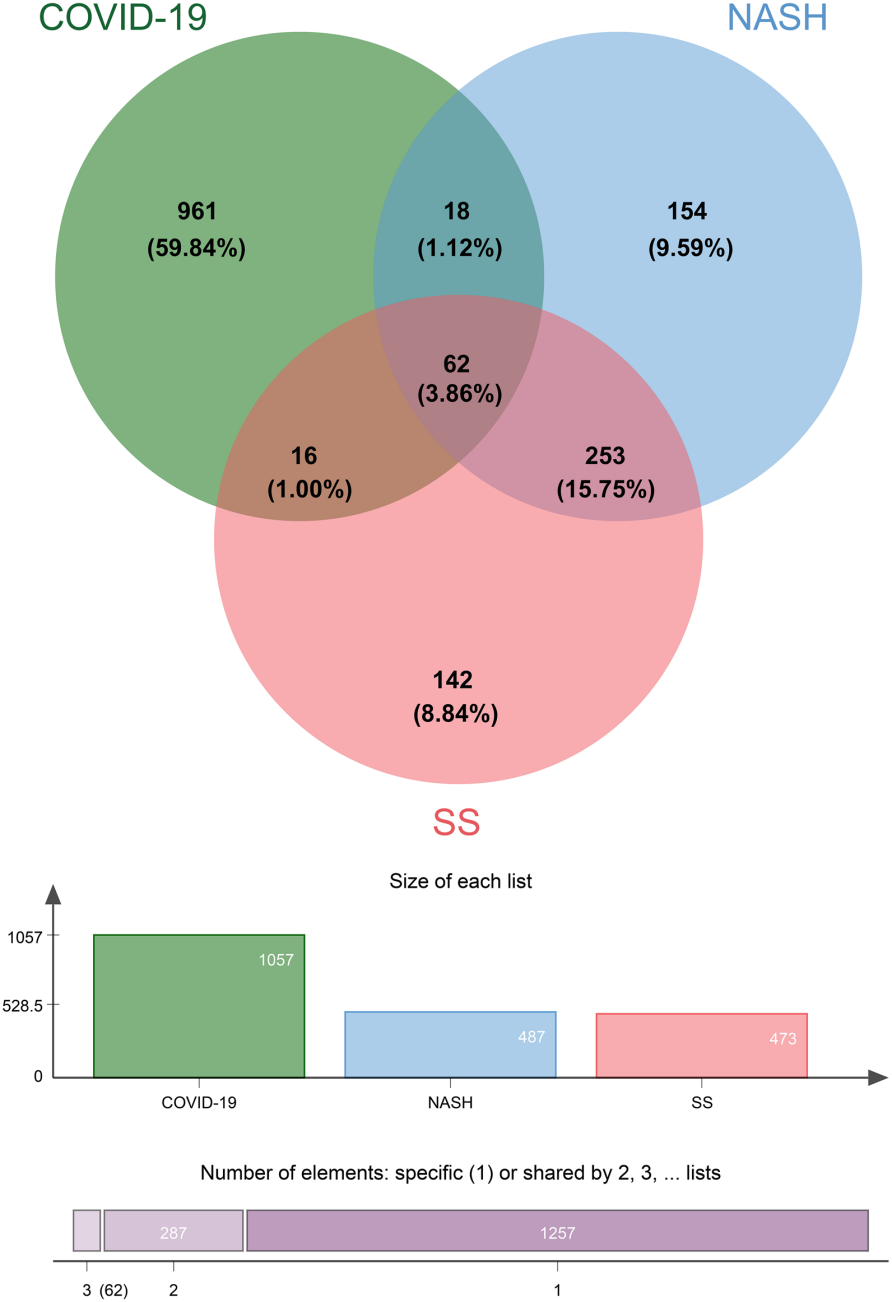
Venn diagram of differential genes for each gene set included in this study. A comprehensive analysis showed that there were 62 common DEGs among COVID-19 (GSE147507), SS (GSE89632), and NASH (GSE89632). DEGs, differential expression genes; COVID-19, coronavirus disease-2019; SS, simple steatosis; NASH, non-alcoholic steatohepatitis

Common DEGs between SS, NASH, and COVID-19 highlight a common molecular pathological basis. Therefore, DEGs were analyzed using RNA-Seq and microarray gene expression datasets from the NCBI GEO database. First, the COVID-19 dataset has 1057 DEGs, including 841 up-regulated genes and 216 down-regulated genes. In addition, we identified 473 DEGs (156 up-regulated and 317 down-regulated) in the SS dataset and 487 DEGs (209 up-regulated and 278 down-regulated) in the NASH dataset. Candidate DEGs were screened based on *P*-values < 0.05 and |logFC|> 1. A cross-comparative analysis of DEGs for the three diseases on the web tool Jvenn identified 62 common DEGs. This set of DEGs was used to complete further studies. The results suggest a connection between COVID-19 and NAFLD, as they share one or more common genes. Figure 2 depicts the results of the comparative analysis of the three datasets and the extracted DEGs, while Additional file 5: Table S4 lists all 62 DEGs.

### Gene ontology (GO) and pathway analysis

**Fig. 3:**
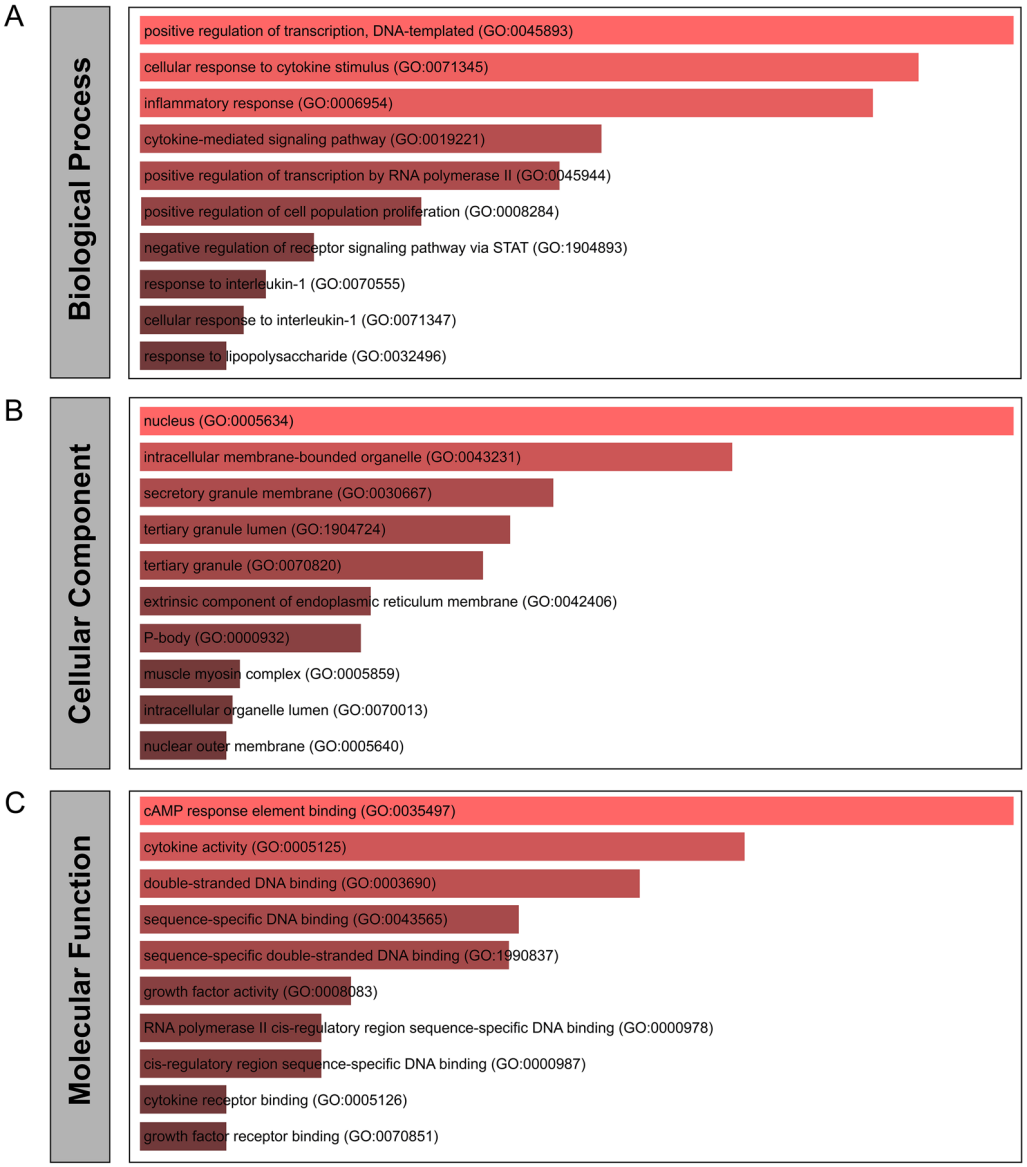
Gene Ontology analysis of the 62 common DEGs among COVID-19, SS, and NASH. **A** biological processes; **B** cellular components; and **C** molecular functions. DEGs, differential expression genes; COVID-19, coronavirus disease-2019; SS, simple steatosis; NASH, non-alcoholic steatohepatitis

GO enrichment analysis was widely used to reflect the relationship between genes and gene ontology terms, while KEGG analysis could reveal the molecular pathways that target genes point. The researchers integrated these two analysis modules in the web tool Enrichr. On the one hand, the GO enrichment analysis allowed us to understand the GO terms associated with common DEGs from three aspects (biological processes (BP), cellular components (CC), and molecular functions (MF)). On the other hand, through the KEGG pathway enrichment analysis, we identified molecular pathways closely associated with common DEGs, which could provide a concrete biological basis for understanding the relationship between diseases. We selected the GO database as the annotation source in the GO enrichment analysis. Sorted by *P*-value, we summarized the top 10 GO terms in BP, CC, and MF categories in Table 2. In addition, the bar chart in Fig. 3 represents the GO analysis based on 62 common DEGs. The DEGs were significantly enriched in positive regulation of transcription, DNA-templated in the subset of BP, in the nucleus in the subset of CC, and cAMP response element binding in the subset of MF.

**Table 2:** Gene Ontology enrichment analysis of common DEGs among COVID-19, SS, and NASH

Category	GO ID	Term	*P-*values	Genes
GO Biological Process	GO:0,045,893	Positive regulation of transcription, DNA-templated	1.06E−08	NFE2;CSF3;JUN;TGFB3;SLC11A1;LIF;FOS;NFKB2;IL6;NR4A3;IL1B;ZC3H12A;BCL3r;MAFF;HIVEP1;NCOA7;ATF3;CREB5
	GO:0,071,345	Cellular response to cytokine stimulus	2.42E−08	SOCS3;CSF3;IL6;SOCS1;CCL20;IL1B;ZC3H12A;LIF;HAS2;IRAK3;FOS;PTGS2
	GO:0,006,954	Inflammatory response	3.59E−08	IL6;TNFAIP6;CCL20;SLC11A1;IL1B;C5AR1;PROK2;PTX3;FOS
	GO:0,019,221	Cytokine-mediated signaling pathway	3.77E−07	SOCS3;CSF3;IL6;SOCS1;CCL20;IL1B;LIF;MAP3K8;IRAK3;FOS;PTGS2;IFIT2
	GO:0,045,944	Positive regulation of transcription by RNA polymerase II	5.43E−07	CSF3;JUN;TGFB3;SLC11A1;LIF;FOS;NFKB2;IL6;NR4A3;ZC3H12A;MAFF;HIVEP1;NCOA7;ATF3
	GO:0,008,284	Positive regulation of cell population proliferation	1.81E−06	CSF3;IL6;NR4A3;TGFB3;IL1B;C5AR1;LIF;PROK2;HAS2;HBEGF
	GO:1,904,893	Negative regulation of receptor signaling pathway via STAT	4.60E−06	SOCS3;SOCS1;BCL3
	GO:0,070,555	Response to interleukin-1	6.97E−06	CCL20;IL1B;ZC3H12A;HAS2;IRAK3
	GO:0,071,347	Cellular response to interleukin-1	8.45E−06	CCL20;IL1B;ZC3H12A;HAS2;MAP3K8;IRAK3
	GO:0,032,496	Response to lipopolysaccharide	9.78E−06	CD274;IL6;SLC11A1;IL1B;ZC3H12A;IRAK3
GO Cellular Component	GO:0,005,634	Nucleus	1.17E−03	ZC3H12A;HIVEP1;ANXA9;IER3;KLF11;NFE2;JUN;PRRX1;GADD45B;IRAK3;SERPINB9;FOS;KIF22;PNRC1;NFKB2;MT1A;NR4A3;BCL3;MAFF;NCOA7;S100P;PADI4;ATF3;APOBEC3A;CREB5
	GO:0,043,231	Intracellular membrane-bound organelle	4.42E−03	ZC3H12A;HIVEP1;ANXA9;IER3;KLF11;NFE2;JUN;PRRX1;GADD45B;TGFB3;IRAK3;SERPINB9;FOS;KIF22;PNRC1;NFKB2;MT1A;NR4A3;BCL3;MAFF;NCOA7;S100P;PADI4;ATF3;APOBEC3A;CREB5
	GO:0,030,667	Secretory granule membrane	1.03E−02	RAB26;SLC11A1;C5AR1;PLAUR
	GO:1,904,724	Tertiary granule lumen	1.26E−02	TNFAIP6;PTX3
	GO:0,070,820	Tertiary granule	1.44E−02	TNFAIP6;SLC11A1;PTX3
	GO:0,042,406	Extrinsic component of endoplasmic reticulum membrane	2.45E−02	ZC3H12A
	GO:0,000,932	P-body	2.56E−02	ZC3H12A;APOBEC3A
	GO:0,005,859	Muscle myosin complex	4.55E−02	MYH11
	GO:0,070,013	Intracellular organelle lumen	4.70E−02	IL6;TNFAIP6;SDC4;PLAUR;PTX3;PTGS2
	GO:0,005,640	Nuclear outer membrane	4.85E−02	PTGS2
GO Molecular Function	GO:0,035,497	cAMP response element binding	3.35E−06	JUN;NR4A3;CREB5
	GO:0,005,125	Cytokine activity	1.58E−05	CSF3;IL6;TGFB3;CCL20;IL1B;LIF
	GO:0,003,690	Double-stranded DNA binding	2.91E−05	KLF11;NFE2;JUN;MAFF;HIVEP1;FOS;BACH2;ATF3;CREB5;NFKB2
	GO:0,043,565	Sequence-specific DNA binding	5.83E−05	KLF11;NFE2;JUN;MAFF;HIVEP1;FOS;BACH2;ATF3;CREB5;NFKB2
	GO:1,990,837	Sequence-specific double-stranded DNA binding	6.19E−05	KLF11;NFE2;JUN;MAFF;HIVEP1;FOS;BACH2;ATF3;CREB5;NFKB2
	GO:0,008,083	Growth factor activity	1.54E−04	CSF3;IL6;LIF;HBEGF
	GO:0,000,978	RNA polymerase II cis-regulatory region sequence-specific DNA binding	1.82E−04	KLF11;NFE2;JUN;NR4A3;PRRX1;MAFF;HIVEP1;FOS;BACH2;ATF3;CREB5;NFKB2
	GO:0,000,987	Cis-regulatory region sequence-specific DNA binding	1.82E−04	KLF11;NFE2;JUN;NR4A3;PRRX1;MAFF;HIVEP1;FOS;BACH2;ATF3;CREB5;NFKB2
	GO:0,005,126	Cytokine receptor binding	3.17E−04	CSF3;IL6;IL1B;LIF
	GO:0,070,851	Growth factor receptor binding	3.17E−04	CSF3;IL6;IL1B;HBEGF

Only the top ten terms for each category were shown. GO, gene ontology; DEGs, DEGs, differential expression genes; COVID-19, coronavirus disease-2019; SS, simple steatosis; NASH, non-alcoholic steatohepatitis

**Fig. 4:**
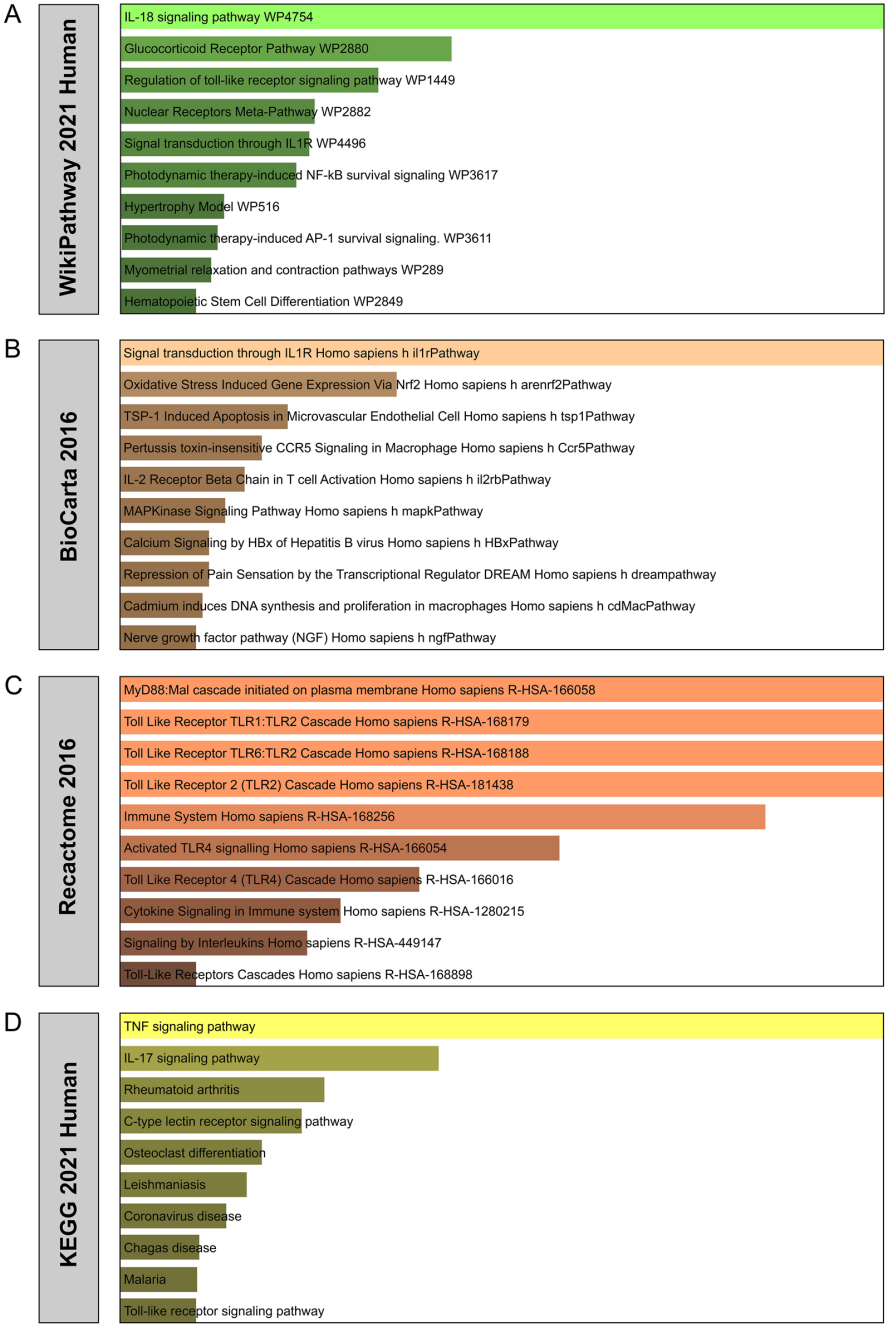
Pathway enrichment analysis of the 62 common DEGs among COVID-19, SS, and NASH. **A** WikiPathway 2021 Human; **B** BioCarta 2016; **C** Reactome 2016, and **D** KEGG 2021 Human. DEGs, differential expression genes; COVID-19, coronavirus disease-2019; SS, simple steatosis; NASH, non-alcoholic steatohepatitis; KEGG, Human Kyoto Encyclopedia of Genes and Genomes

This study gathered the most impacted pathways of the common DEGs among NAFLD, and COVID-19 from four sources, including KEGG, WikiPathways, Reactome, and BioCarta. In particular, KEGG pathway analysis had been widely used for the functional identification of genes and their interaction networks. Table 3 lists the top pathways obtained from the selected datasets. For a more accurate illustration, the bar chart in Fig. 4 showed the pathway enrichment analysis. GO, KEGG pathway analysis, gene counts, and gene symbols are also displayed in Additional file 6: Table S5 and Additional file 7: Table S6, respectively.

**Table 3:** Pathway enrichment analysis of common DEGs among COVID-19, SS, and NASH

Category	Pathways	*P* - values	Genes
WikiPathway 2021 Human	IL-18 signaling pathway WP4754	8.60E−14	JUN;SDC4;CCL20;RGS16;FOS;PTGS2;NFKB2;SOCS3;IL6;IL1B;ZC3H12A;PTX3;ATF3;IER3
	Glucocorticoid receptor pathway WP2880	2.00E−09	JUN;GADD45B;CCL20;SERPINB9;S100P;PTGS2;NFKB2
	Regulation of toll-like receptor signaling pathway WP1449	1.10E−08	JUN;IL6;SOCS1;IL1B;MAP3K8;IRAK3;FOS;NFKB2
	Nuclear receptors meta-pathway WP2882	4.78E−08	JUN;GADD45B;CCL20;IL1B;MAFF;SERPINB9;S100P;PTGS2;HBEGF;NFKB2
	Signal transduction through IL1R WP4496	5.39E−08	JUN;IL6;TGFB3;IL1B;IRAK3
	Photodynamic therapy-induced NF-kB survival signaling WP3617	7.34E−08	IL6;BCL2A1;IL1B;PTGS2;NFKB2
	Hypertrophy Model WP516	3.91E−07	NR4A3;CYR61;ATF3;HBEGF
	Photodynamic therapy-induced AP-1 survival signaling. WP3611	4.62E−07	JUN;IL6;BCL3;FOS;HBEGF
	Myometrial relaxation and contraction pathways WP289	5.27E−07	JUN;IL6;IL1B;RGS16;MAFF;FOS;ATF3
	Hematopoietic stem cell differentiation WP2849	7.50E−07	NFE2;CSF3;IL6;IL1B;FOS
BioCarta 2016	Signal transduction through IL1R Homo sapiens h il1rPathway	1.25E−09	JUN;IL6;TGFB3;IL1B;IRAK3;FOS
	Oxidative Stress induced gene expression Via Nrf2 Homo sapiens h arenrf2Pathway	2.24E−05	JUN;MAFF;FOS
	TSP-1 Induced apoptosis in microvascular endothelial cell Homo sapiens h tsp1Pathway	1.97E−04	JUN;FOS
	Pertussis toxin-insensitive CCR5 signaling in macrophage Homo sapiens h Ccr5Pathway	3.36E−04	JUN;FOS
	IL-2 receptor beta chain in T cell activation Homo sapiens h il2rbPathway	4.72E−04	SOCS3;SOCS1;FOS
	MAPKinase signaling pathway Homo sapiens h mapkPathway	6.99E−04	JUN;MAP3K8;FOS
	Calcium signaling by HBx of Hepatitis B virus Homo sapiens h HBxPathway	9.67E−04	JUN;FOS
	Repression of pain sensation by the Transcriptional Regulator DREAM Homo sapiens h dreampathway	9.67E−04	JUN;FOS
	Cadmium induces DNA synthesis and proliferation in macrophages Homo sapiens h cdMacPathway	0.001103311	JUN;FOS
	Nerve growth factor pathway (NGF) Homo sapiens h ngfPathway	0.001247926	JUN;FOS
Reactome 2016	MyD88:Mal cascade initiated on plasma membrane Homo sapiens R-HSA-166058	4.01E−07	JUN;SOCS1;MAP3K8;IRAK3;FOS;NFKB2
	Toll like receptor TLR1:TLR2 cascade Homo sapiens R-HSA-168179	4.01E−07	JUN;SOCS1;MAP3K8;IRAK3;FOS;NFKB2
	Toll like receptor TLR6:TLR2 cascade Homo sapiens R-HSA-168188	4.01E−07	JUN;SOCS1;MAP3K8;IRAK3;FOS;NFKB2
	Toll like receptor 2 (TLR2) cascade Homo sapiens R-HSA-181438	4.01E−07	JUN;SOCS1;MAP3K8;IRAK3;FOS;NFKB2
	Immune System Homo sapiens R-HSA-168256	6.13E−07	CD274;CSF3;JUN;SLC11A1;C5AR1;LIF;IRAK3;FOS;KIF22;IFIT2;NFKB2;SOCS3;IL6;SOCS1;IL1B;MAP3K8;IER3;HBEGF
	Activated TLR4 signaling Homo sapiens R-HSA-166054	1.28E−06	JUN;SOCS1;MAP3K8;IRAK3;FOS;NFKB2
	Toll Like Receptor 4 (TLR4) cascade Homo sapiens R-HSA-166016	2.12E−06	JUN;SOCS1;MAP3K8;IRAK3;FOS;NFKB2
	Cytokine Signaling in Immune system Homo sapiens R-HSA-1280215	2.81E−06	SOCS3;CSF3;IL6;SOCS1;IL1B;LIF;MAP3K8;IRAK3;IFIT2;HBEGF;NFKB2
	Signaling by interleukins Homo sapiens R-HSA-449147	3.16E−06	SOCS3;CSF3;IL6;IL1B;LIF;MAP3K8;IRAK3;HBEGF;NFKB2
	Toll-like receptors cascades Homo sapiens R-HSA-168898	4.71E−06	JUN;SOCS1;MAP3K8;IRAK3;FOS;NFKB2
KEGG 2021 Human	TNF signaling pathway	4.11E−14	SOCS3;JUN;IL6;CCL20;IL1B;BCL3;LIF;MAP3K8;FOS;PTGS2;CREB5
	IL-17 signaling pathway	1.61E−08	CSF3;JUN;IL6;CCL20;IL1B;FOS;PTGS2
	Rheumatoid arthritis	4.28E−07	JUN;IL6;TGFB3;CCL20;IL1B;FOS
	C-type lectin receptor signaling pathway	8.30E−07	JUN;IL6;IL1B;BCL3;PTGS2;NFKB2
	Osteoclast differentiation	2.68E−06	SOCS3;JUN;SOCS1;IL1B;FOS;NFKB2
	Leishmaniasis	4.04E−06	JUN;TGFB3;IL1B;FOS;PTGS2
	Coronavirus disease	7.38E−06	CSF3;JUN;IL6;IL1B;C5AR1;FOS;HBEGF
	Chagas disease	1.61E−05	JUN;IL6;TGFB3;IL1B;FOS
	Malaria	1.73E−05	CSF3;IL6;TGFB3;IL1B
	Toll like receptor signaling pathway	1.76E−05	JUN;IL6;IL1B;MAP3K8;FOS

Only the top ten pathways for each category were shown. DEGs, DEGs, differential expression genes; COVID-19, coronavirus disease-2019; SS, simple steatosis; NASH, non-alcoholic steatohepatitis; KEGG, Human Kyoto Encyclopedia of Genes and Genomes

### Hub gene extraction and submodule analysis

**Fig. 5:**
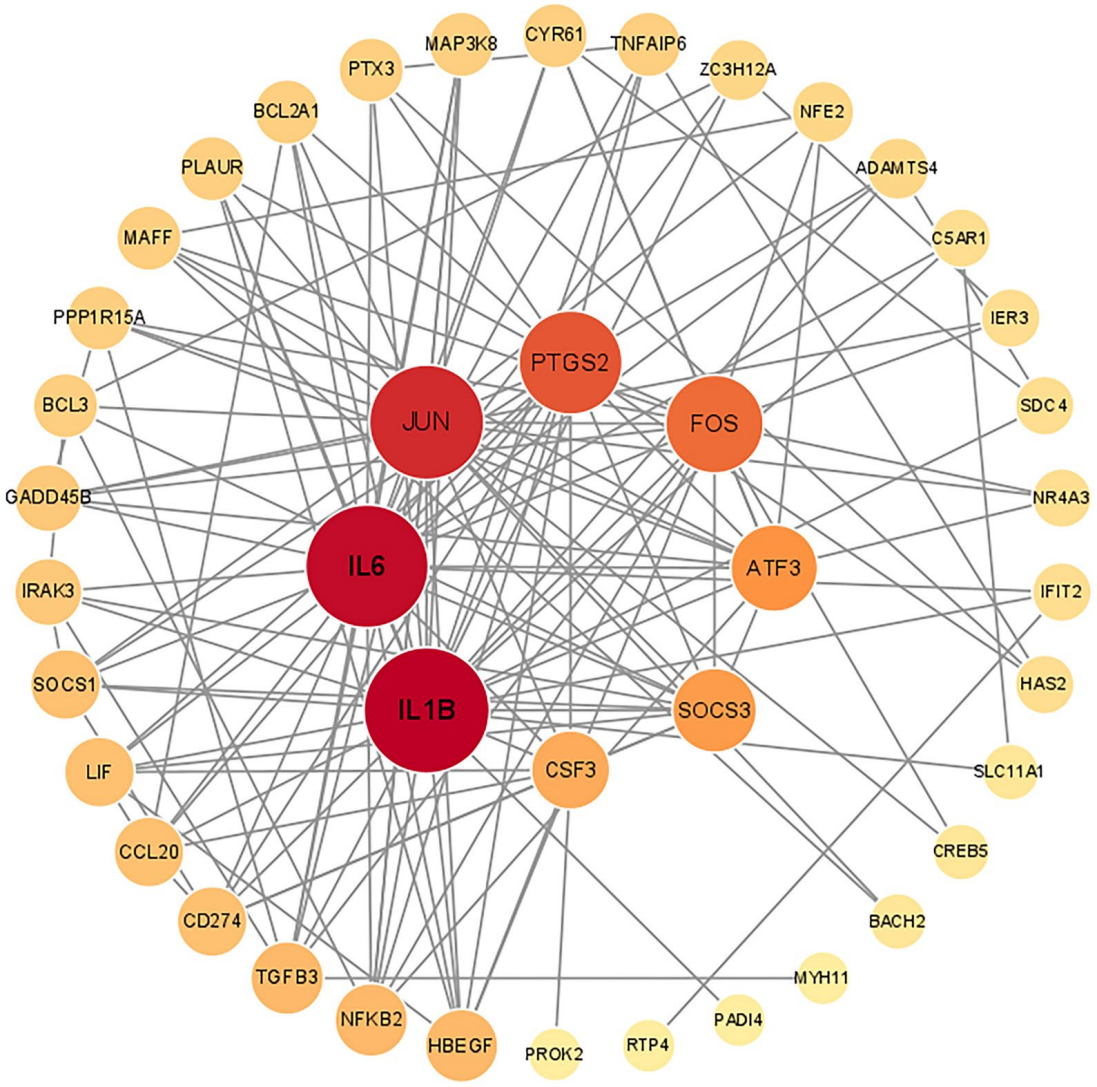
PPI network of the common DEGs among COVID-19, SS, and NASH. The circle nodes represent DEGs and edges represent the interactions between nodes. The PPI network was generated using String and visualized in Cytoscape. All genes were arranged in descending order of degree. PPI, protein–protein interactions; DEGs, differential expression genes; COVID-19, coronavirus disease-2019; SS, simple steatosis; NASH, non-alcoholic steatohepatitis

**Fig. 6:**
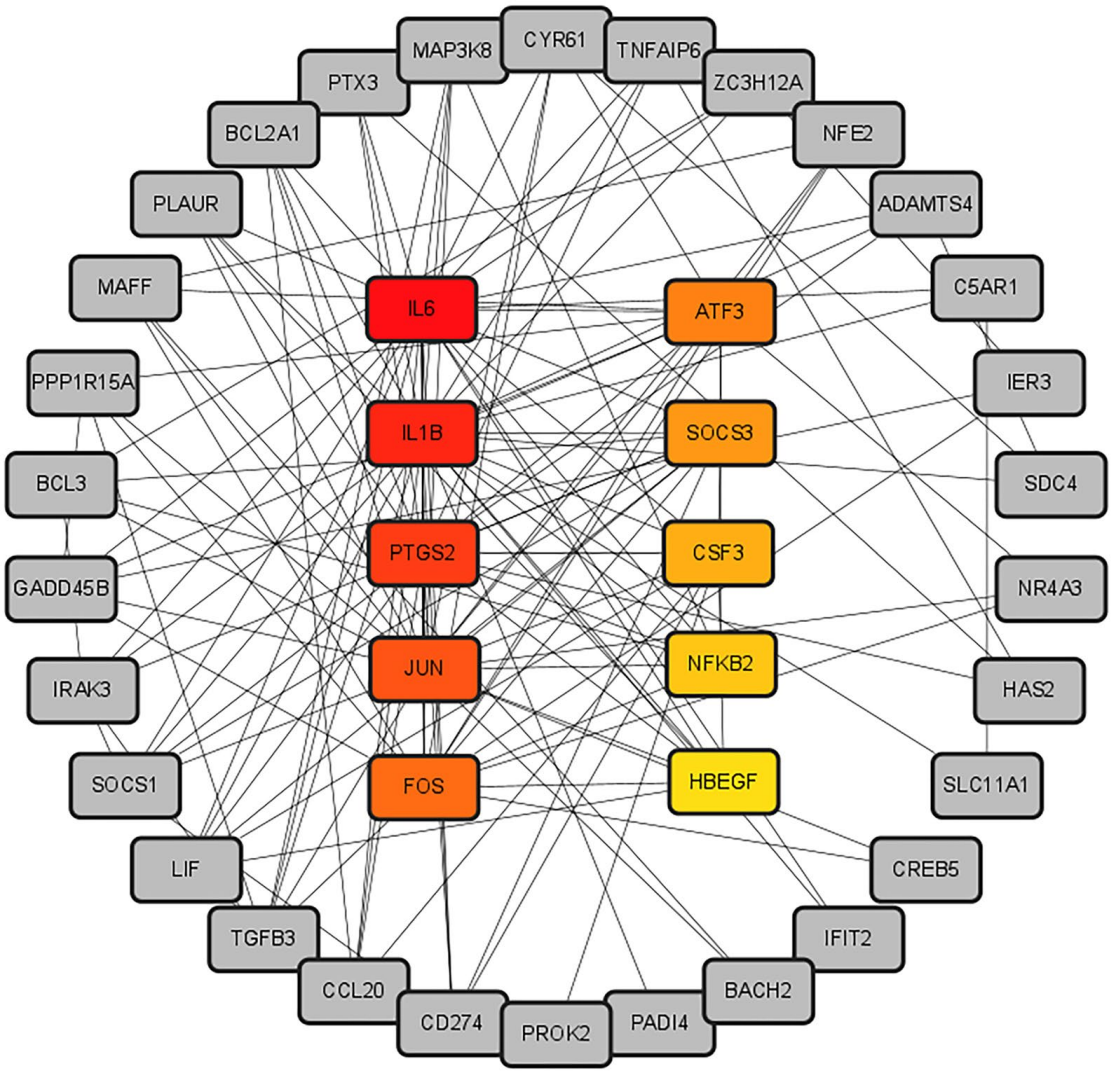
The hub gene was identified from the PPI network using the Cytohubba plug-in Cytosacpe. The colored nodes represent the highlighted top 10 hub genes and their interactions with other molecules. PPI, protein–protein interactions

We imported the PPI network from STRING into Cytoscape for visualization to show the interaction relationships and paths between common DEGs. The PPI network of common DEGs consisted of 42 nodes and 148 edges, as shown in Fig. 5. Additional file 8: Table S7 summarizes the topological characteristics of PPI interaction networks obtained from STRING, such as degree, mesoscopic centrality, stress centrality, and proximity centrality. Meanwhile, genes with high connectivity in PPI networks were considered network hub genes. Based on the analysis results of the Cytohubba plug-in in Cytoscape, we listed the top 10 (16.13%) common DEGs as the most influential genes. The hub genes were IL6, IL1B, PTGS2, JUN, FOS, ATF3, SOCS3, CSF3, NFKB2, and HBEGF, ranked by importance scores calculated by the Cytohubba plug-in. We did ROC analysis of the above ten hub genes in cohorts of SS, NASH, and COVID-19 patients, respectively, and the results are demonstrated in Additional file 1: Figure S1. Firstly, as shown in Additional file 1: Figure S1A, the AUC values of all eight hub genes were more significant than 0.9 in the SS cohort except for IL1B and CSF3. In the cohort NASH (Additional file 1: Fig. S1B), the AUC values of all hub genes were greater than 0.9, except for NFKB2. In the COVID-19 cohort (Additional file 1: Fig. S1C), IL6, PTSG2, JUN, ATF3, SOCS3, CSF3, and NFKB2 had AUC values greater than 0.7. Ten hub genes were included in the logistic regression analysis in the SS and NASH cohorts and modeled with an AUC value of 1. Similarly, in the COVID-19 cohort, ten genes were included in the logistic regression analysis with a modeled AUC value of 0.947. The above hub genes might be potential biomarkers for NAFLD and COVID-19, leading to new therapeutic strategies for the diseases we studied. With the help of the Cytohubba, we also constructed a submodule network (Fig. 6) to gain more insight into the linkage between hub genes. The interactions between hub genes and other common DEGs are shown in Fig. 6.

### Regulatory network of DEGs-related TFs and miRNAs

**Fig. 7:**
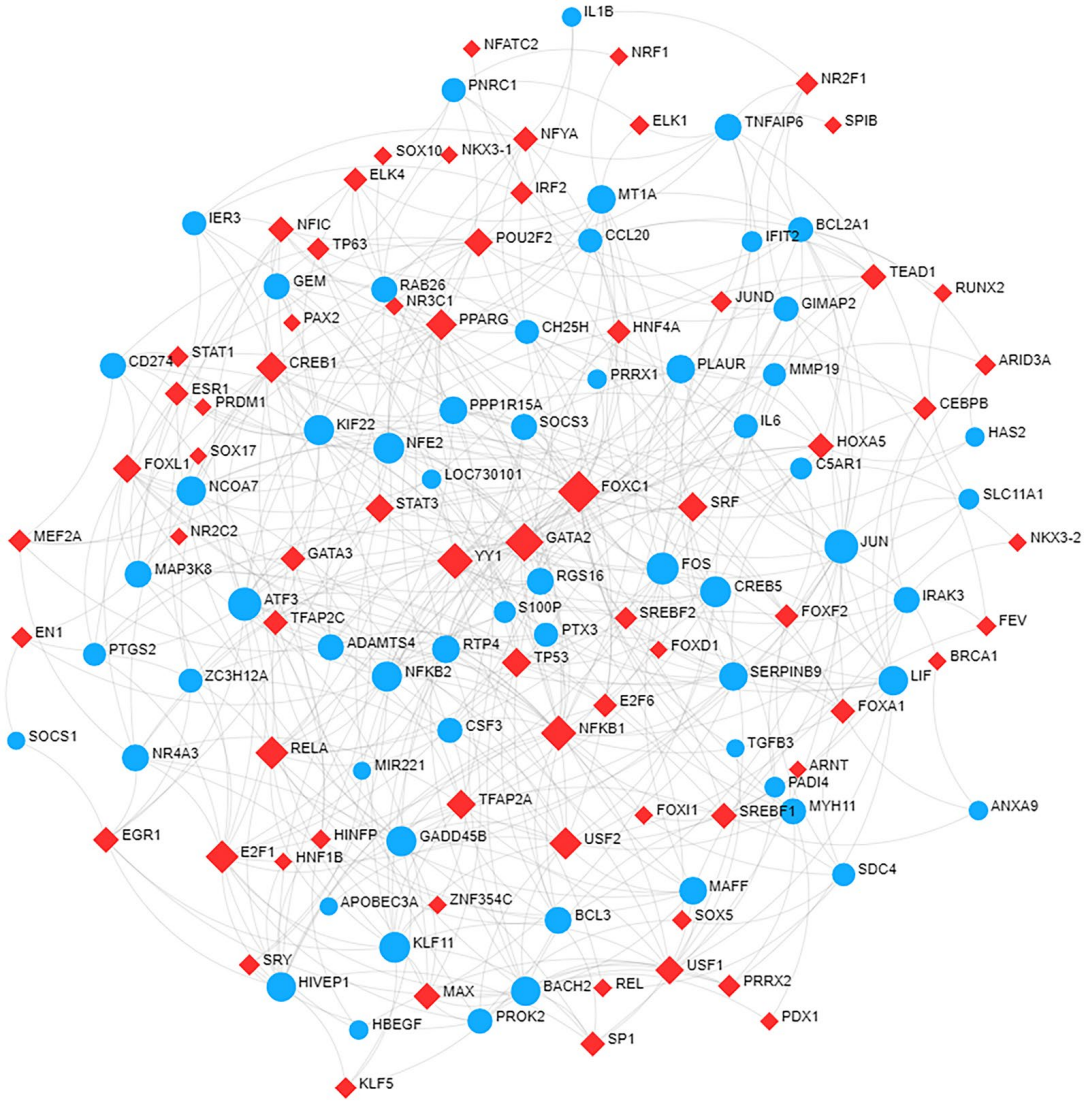
The cohesive regulatory interaction network of DEGs–TFs obtained from the NetworkAnalyst. The square nodes represented TFs, and gene symbols interact with TFs as circle nodes. DEGs, differential expression genes; TFs, transcription factors

**Fig. 8:**
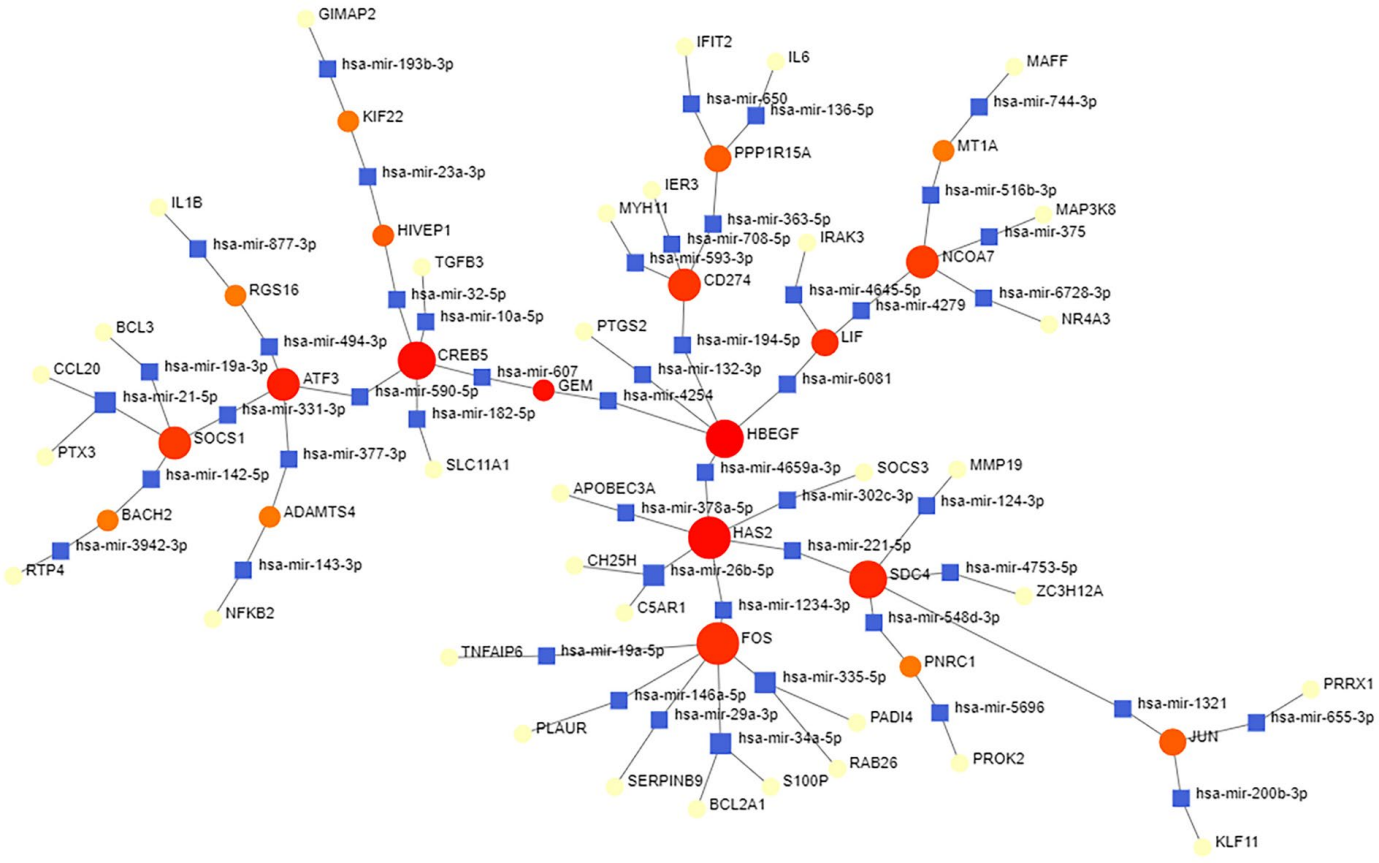
The interconnected regulatory interaction network of DEGs–miRNAs. The circle node indicates miRNAs and the gene symbols interact with miRNAs in the shape of a square. DEGs, differential expression genes

To detect significant changes occurring at the transcriptional level and gain insight into the regulatory molecules of hub DEGs, we analyzed the regulatory connectivity network between TFs, miRNAs, and common DEGs. We found 69 TFs and 50 miRNAs regulatory signals regulating common DEGs, suggesting substantial interference between them. Figure 7 details the regulatory relationships between TFs and common DEGs. The analysis showed that FOXC1 was the most prominent TF in this network. In addition, Fig. 8 concisely summarizes the interactions between miRNA regulators and common DEGs. Additional file 9: Table S8 and Additional file 10: Table S9 summarize the data used to construct the regulatory networks of TFs–DEGs and miRNAs–DEGs.

### Potential drugs

Assessing protein–drug interactions was extremely important to understand the structural features of molecules with potential therapeutic targets. We used common DEGs as potential drug targets, identified various compounds with statistical differences using Enrichr, and extracted the 10 most promising compounds for development based on *P* values. The above analysis used the DsigDB database as an annotation source. These potential drugs were suggested for common DEGs used to treat the diseases covered in this study. Table 4 summarizes the effective drugs in the DSigDB database for common DEGs.

**Table 4:** Potential drugs targeting common DEGs among COVID-19, SS, and NASH

Name	*P* - values	Molecular formula	Molecular weight	Structure formula
Digoxin HL60 UP	2.20E−24	C_41_H_64_O_14_	780.9	
8-Azaguanine PC3 UP	1.24E−22	C_4_H_4_N_6_O	152.11	
Ciclopirox HL60 UP	3.00E−21	C_12_H_17_NO_2_	207.27	
Ouabain HL60 UP	3.52E−21	C_29_H_44_O_12_	584.7	
Etoposide HL60 UP	6.43E−21	C_29_H_32_O_13_	588.6	
Menadione HL60 UP	7.85E−21	C_11_H_8_O_2_	172.18	
Cephaeline HL60 UP	1.03E−19	C_28_H_38_N_2_O_4_	466.6	
ZINC CTD	5.39E−19	Zn	65.4	Zn
Cicloheximide PC3 UP	2.33E−18	C_15_H_23_NO_4_	281.35	
Pyrvinium HL60 UP	5.58E−18	C_26_H_28_N_3_^+^	382.5	

The top ten drugs are shown. DEGs, DEGs, differential expression genes; COVID-19, coronavirus disease-2019; SS, simple steatosis; NASH, non-alcoholic steatohepatitis

### Disease–gene network

**Fig. 9:**
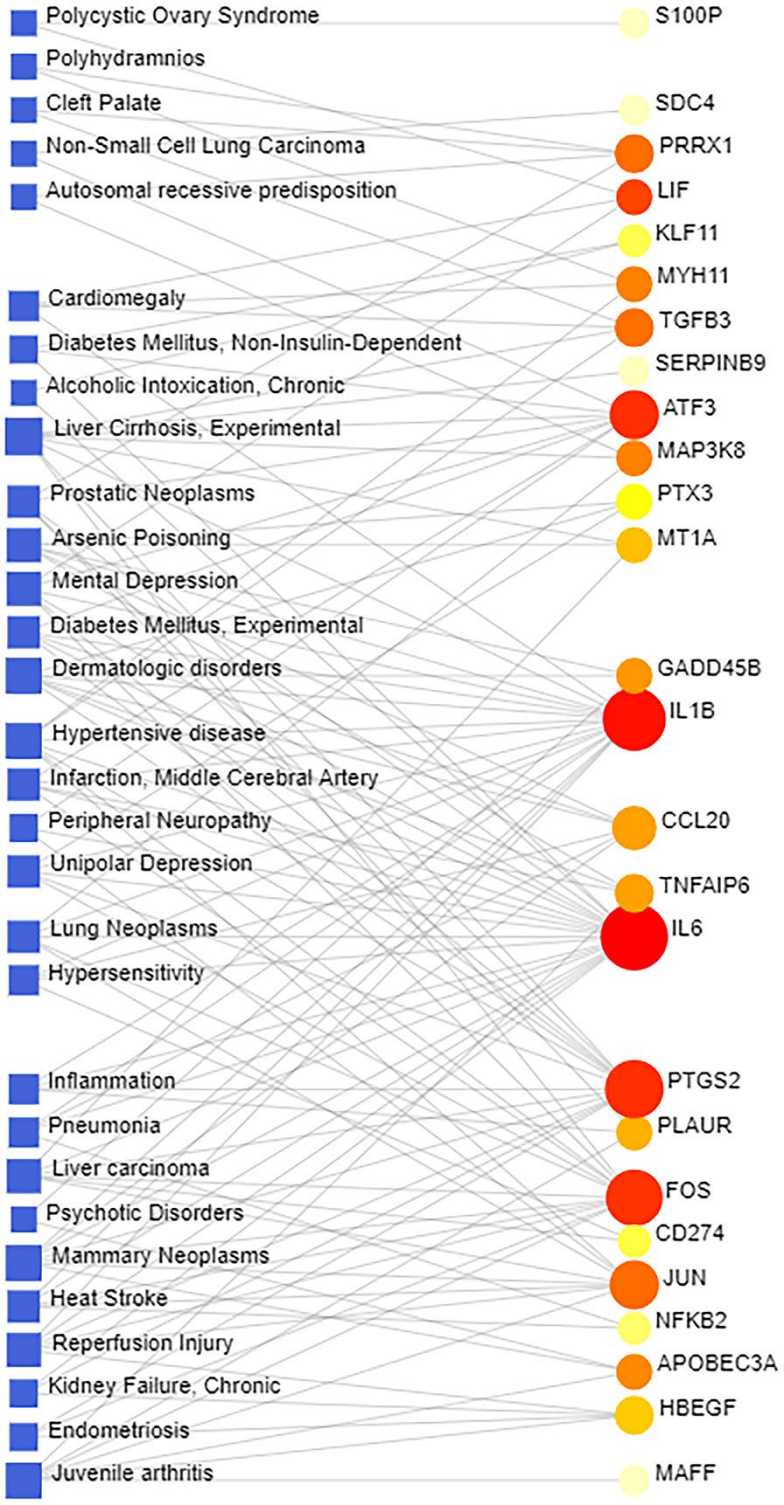
The gene–disease association network represents diseases associated with common DEGs. The disease represented by the square node and also its subsequent gene symbols were defined by the circular node

Correlation analysis of different diseases or abnormal states presupposed that they shared a similar genetic basis or common DEGs. Revealing the link between genes and diseases was an essential component of designing disease treatment strategies. Using disease correlation analysis of common DEGs, we found that cirrhosis, hypertension, juvenile arthritis, skin diseases, and breast tumors had the highest correlations with the common DEGs we reported. Figure 9 illustrates the relationship between common DEGs and diseases.

## Discussion

COVID-19 continues to be prevalent worldwide and significantly affects people's lives [1]. Given the high prevalence of NAFLD in the population, researchers are interested in assessing the potential impact of COVID-19 on NAFLD. Several studies have suggested that the presence of NAFLD may affect the prognosis of patients with COVID-19 [6, 28–30]. NAFLD may increase the risk of hospitalization and the incidence of severe disease in patients with COVID-19 [31, 32]. SS and NASH are manifestations of NAFLD at different stages of the disease. Studies on the common molecular mechanisms between NAFLD and COVID-19 are still lacking. In this study, we developed a network-based systems biology approach to study gene expression patterns in patients with NAFLD and COVID-19 and identified potential biomarkers and therapeutic agents. Expression profiling using high-throughput sequencing datasets has become an essential part of systems biology research. Our transcriptome analysis of NAFLD and COVID-19 revealed that 62 common DEGs showed similar expression patterns in both diseases. The structure of GO has evolved with new scientific discoveries. We performed three types of GO analysis using Enrichr.

For biological processes, common DEGs were mainly enriched in positive regulation of transcription and cytokine-mediated responses (Table 2). Positive regulation of transcription, DNA-templated (GO:0045893), and cellular response to cytokine stimulus (GO:0071345) were among the top GO terms. Among the occurrence and progression of NAFLD and COVID-19, the release of cytokines and the activation of inflammatory response played essential roles in the process [33]. From the synthesis of cytokines to the proliferation of immune cells, DNA-templated transcription was the basis of all biological processes. Cytokines were a broad and loose category of small proteins essential in cell signaling. As immunomodulating agents, cytokines were involved in autocrine, paracrine, and endocrine signaling. “Cytokine storm” was often observed in severe COVID-19 and could lead to multiple organ failures, including severe liver damage [34, 35]. A recent study demonstrated that IL-6 was probably the main cytokine orchestrating this inflammatory response leading to liver damage [36]. Given that systemic inflammatory response syndrome (SIRS) induces inflammation in multiple tissues, including the liver, it is reasonable to assume that it may further aggravate a pre-existing inflammatory state, like the one observed in NASH [37]. This hypothesis is further supported by the fact that several of the elevated cytokines in the circulation of patients with severe COVID-19 [37, 38], such as CCL2 and TNF-α, have a crucial role in the development and progression of NAFLD by recruiting inflammatory cells in the liver or by regulating hepatocyte apoptosis [39]. In the part of CC, the nucleus (GO:0005634) and intracellular membrane-bound organelle (GO:0043231) were two significant GO CC terms. According to the annotation results of GO:0005634 and GO:0043231, we found that the enrichment results of CC were closely related to the positive regulation of transcription. For the SARS-CoV, the membrane protein, spike glycoprotein, and envelope protein are produced by the ribosome and implanted into the endoplasmic reticulum membrane during the replication of SARS-CoV-2 [40]. A significant enhancement of transcription accompanies this process. In the MF analysis, cAMP response element binding (GO:0035497) and cytokine activity (GO:0005125) were two top GO terms.

KEGG pathway analysis is the primary method to assess an organism's higher-level systematic responses to internal changes [41]. Sixty-two common DEGs were identified to find a similar pathway for NAFLD and COVID-19. The top 10 KEGG pathways were: TNF signaling pathway, IL-17 signaling pathway, rheumatoid arthritis, C-type lectin receptor signaling pathway, osteoclast differentiation, leishmaniasis, coronavirus disease, asthma, Chagas disease, malaria, and Toll like receptor signaling pathway. There was evidence that the central mediator of cytokine storm may be TNF. This cytokine was known to be involved in activated blood clotting, lung damage, insulin resistance, heart failure, and other conditions [42]. Observational data from patients already on anti-TNF therapy show a reduced rate of COVID-19 poor outcomes and death compared with other immune-suppressing therapies [43].

A PPI network was built using the common DEGs among NAFLD and COVID-19. The hub gene may be an important drug target or biomarker for COVID-19 and is associated with the progression of NAFLD. According to the MCC method, the top 10 hub genes included: IL6, IL1B, PTGS2, JUN, FOS, ATF3, SOCS3, CSF3, NFKB2, and HBEGF.

IL-6 is a pleiotropic cytokine that regulates the immune system and the inflammatory response and affects hematopoiesis, metabolism, organ development, and cancer growth [44]. In COVID-19, IL-6 is believed to drive multi-organ injury, the most severe form of the illness [45, 46]. There was sufficient evidence that in patients with Covid-19, IL-6 levels were significantly elevated and associated with adverse clinical outcomes [47]. Patra et al. found that the SARS-CoV-2 spike protein can trigger an angiotensin II type 1 (AT1) receptor-mediated signaling cascade that ultimately increases IL-6 release [48]. In infected lung tissue, high levels of IL-6 activate inflammation-related cells. At the same time, localized SARS-CoV-2 replication leads to increased cytokine production, ultimately leading to organ damage [49–51]. In addition, IL-6 can also aggravate the damage to systemic organ function by inducing apoptosis of lymphocytes [52, 53] and participating in coagulation dysfunction associated with COVID-19 [54]. To clarify the co-action of IL-6 in patients with COVID-19 and NAFLD, we extensively searched relevant literature.

Recently, a multicenter retrospective study by Gao et al. showed that serum IL-6 levels were significantly higher in NAFLD patients after infection with SARS-CoV-2 than in non-NAFLD patients. Furthermore, patients with NAFLD had an approximately 2.6-fold higher risk of severe COVID-19 than those without NAFLD [55]. McConnell et al. observed high levels of IL-6 and its circulating receptor complexed to induce inflammatory signaling in COVID-19 patients [56]. They believe that the IL-6 signaling complex causes detrimental changes in liver sinusoidal endothelial cells and may promote blood clotting and lead to liver damage. Although we currently do not know the exact mechanism by which IL-6 is associated with NAFLD and COVID-19. The presence of NAFLD may represent a chronic low-grade inflammatory state with compromised immune responses, often accompanied by an accumulation of cytokines, which increases the susceptibility of NAFLD patients to COVID-19. In addition, COVID-19 often triggers cytokine storms, and excessive immune activation may cause abnormal immune cell recruitment in the liver of NAFLD patients, thereby aggravating liver damage.

Like IL-6, IL-1, a pleiotropic cytokine, plays a vital role in biological processes such as immunity and inflammation [57]. IL-1B is the most studied member of IL-1 family because of the function of IL-1B in regulating autoinflammatory diseases [58]. Multiple studies have shown that IL-1 levels are elevated in patients infected with SARS-CoV-2 [59]. IL-1B can promote bronchial and alveolar inflammation in patients with lung tissue damage [60]. In addition, IL-1B can stimulate hepatocytes to produce acute-phase proteins [61]. Mridha et al. found that an increase in IL-1B is associated with NASH progression. By inhibiting the expression of IL-1B in a mouse model, the numbers of macrophages and neutrophils in the liver were reduced, and liver fibrosis was improved [62]. The association between IL-6 and IL-1β in NAFLD patients infected with SARS-CoV-2 remains to be further elucidated.

PTSG2, also known as cyclooxygenase two or COX-2, is an enzyme encoded in the human body by the PTGS2 gene [63]. Studies have shown that the cyclooxygenase family plays an essential role in COVID-19 [64]. SARS-CoV-2 induced COX-2 upregulation in various human cell cultures and mouse models [65]. Furthermore, upregulation of COX-2 may increase mortality and morbidity in COVID-19 patients. High levels of COX-2 lead to depletion of the endogenous antiviral compound arachidonic acid, making individuals more susceptible to COVID-19 infection [66]. Activation of COX-2 has been reported to be involved in the pathogenesis of different liver diseases, including NAFLD. NAFLD is considered to be the hepatic manifestation of metabolic syndrome. Some reports suggest that PGs may promote hepatocyte lipid accumulation [67]. During the onset of metabolic syndrome and type 2 diabetes, activation of COX-2 can trigger opposite exacerbating effects on the progression of NASH [68].

Homo- or heterodimers of JUN, FOS, and ATF constitute AP-1 [69]. Cytokine expression in human airway epithelial cells is regulated by AP-1 [70]. Early studies have confirmed that JUN, FOS, and ATF3 are up-regulated in COVID-19 patients and correlated with the severity of COVID-19 [71, 72]. Cytokine storms caused by COVID-19 can be prevented by reducing JUN and FOS expression [73]. At the same time, some studies have shown that JUN and FOS are closely related to the formation of hepatocellular carcinoma [74]. ATF3 is overexpressed in fatty liver and may play an essential role in the occurrence and development of oxidative stress-mediated hepatic steatosis, and ATF3 silencing in vivo may be a potentially important target for the prevention of NAFLD [75].

The expression of SOCS3 downregulates the JAK2/STAT3 pathway to promote macrophage polarization, which plays a crucial role in lung inflammation [76]. Antagonists of SOCS3 were proposed by Johnson et al. as potential drugs for the treatment of COVID-19 [77]. These findings suggested an essential role of SOCS3 in COVID-19 disease progression. Meanwhile, Nishika Sahini et al. found that SOCS3 is closely related to NAFLD [78].

CSF3 was the most up-regulated gene after SARS-CoV2 infection [79]. Fang et al. confirmed that elbasvir and ritonavir can significantly inhibit CSF3 protein expression [80], indicating that CSF3 is a potential target for the treatment of COVID-19. However, only a few studies have linked CSF3 to NAFLD progression [81].

NF-kB signaling pathway genes (NFKBIA, NFKB1, RELA, NFKB2) were up-regulated in COVID-19 patients [82, 83]. Furthermore, Leng et al. found that the non-canonical NF-κB/NFKB2 pathway was markedly activated in the lungs of COVID-19 patients, leading to chemokine and cytokine production and lymphoid organogenesis [84]. Activation of the non-canonical NF-κB/NFKB2 pathway has never been reported in cytokine storms caused by other respiratory viruses such as influenza. Therefore, they suggest that the non-canonical NF-κB/NFKB2 pathway may be a potential drug design target [84, 85]. Malik et al. found a significant elevation of NFKB2 in fatty liver of mice fed a high-fat diet [86]. The role of NFKB2 in NAFLD remains to be further studied.

T. Venkataraman et al. found that upregulation of HBEGF leads to an aggravation of pulmonary fibrosis after SARS-CoV infection [87]. In addition, HBEGF also played an essential role in metabolic diseases. Kim et al. suggested that HBEGF was a positive stimulator of hepatic very-low-density lipoprotein (VLDL) production that may cause hypertriglyceridemia under obesity and over-nutrition [88]. Studies have also shown that targeting HBEGF can be a practical approach to prevent and reverse vascular and liver inflammation, as demonstrated in mouse models. If our biological view of COVID-19 is confirmed, these hub genes could be considered robust biomarkers and new drug targets.

We also found that common DEGs interacted with TFs and miRNAs in various diseases. TFs regulate target gene transcription and expression, whereas miRNAs impact post-transcriptional genes and influence organism development. FOXC1, GATA2, YY1, NFKB1, E2F1, RELA, USF2, PPARG, CREB1, LIF, and BACH2 are only a few of the TFs linked to NAFLD. Mir-335-5p, mir-363-5p, mir-34a-5p, and mir-29a-3p were found to be related with NAFLD using DEGs–miRNA visualization. Fan et al., through functional verification, showed that miR-335-5p prevented NASH occurrence [89]. Furthermore, mir-363-5p was linked to NAFLD, and HCC patients with reduced mir-363-5p expression had a greater overall survival rate [90]. For risk stratification of NASH patients, Harrison et al. devised a non-invasive diagnostic approach containing mir-34a-5p [91]. GAS5 operated as a miR-29a-3p sponge, promoting NAFLD progression by targeting the miR-29a-3p/NOTCH2 axis, according to Cui et al. [92]. We also found five miRNAs linked with COVID-19 (mir-26b-5p, mir-21-5p, mir-34a-5p, mir-4659a-3p, mir-142-5p). Mir-21-5p was found by Tang et al. as a potential contribution to disease pathogenesis, a biomarker, and a candidate therapeutic target for severe COVID-19 [93].

To anticipate the connection of common DEGs with different disorders, we used a gene–disease (GD) analysis. The experiments' findings revealed several disorders linked to COVID-19 and NAFLD. Liver cirrhosis was discovered in the visible disease network. SARS-CoV-2 has a significant tropism for the liver and biliary tract, according to studies, and many COVID-19 patients have liver function impairment or a worsening of chronic liver disease during the disease process [6, 94]. Genes associated with psychiatric disorders, such as depression, had also been identified. According to recent research, people with depression and schizophrenia were at a higher risk of developing SARS-CoV-2 [95, 96]. In addition, we discovered that endometriosis was linked to several genes. Endometriosis is a disease that is linked to chronic stress. The COVID-19 pandemic may cause post-traumatic stress disorder, psychological distress, sadness, and anxiety [97]. Using network analysis, we discovered dermatologic disorders as well. It had been reported that the side effects of SARS-CoV-2 infection could cause more severe pruritus [98]. COVID-19 is also associated with most malignancies, including breast tumors, prostate tumors, liver tumors, and lung tumors. Due to the severity of the disease and the weakened immune system, cancer patients were at higher risk of developing severe COVID-19 and death [99]. Cardiovascular injury could be induced by various factors, including systemic inflammation and ischemia [100]. Multiple common DEGs related to hypertension, diabetes, and cardiovascular disease progression were retrieved in this study. According to recent findings, COVID-19 patients die from various conditions, including chronic lung disease, diabetes, cardiovascular disease, and hypertension [101, 102].

Several medicines have been utilized to combat COVID-19 so far. remdesivir [103], favipiravir [104], and molnupiravir [105] are a few examples. Furthermore, a clinical investigation found that hydroxychloroquine had a substantial benefit in COVID-19 patients, which was improved by azithromycin [106]. A meta-analysis, however, revealed that combining hydroxychloroquine and azithromycin increased mortality [107]. Identifying new medications and chemicals to treat SARS-CoV-2 infection is unavoidable in the context of the COVID-19 pandemic.

Using network-based analysis, we identified compounds that could act on common DEGs. Digoxin and ouabain, two FDA-approved heart disease medications, exhibit antiviral action against numerous coronaviruses. Cho et al. demonstrated that digoxin and ouabain prevented more than 99 percent of SARS-COV-2 replication, leading to viral suppression at the viral life cycle's entrance stage [108]. Furthermore, investigations have revealed that ouabain and digoxin blocked SARS-CoV-2 spike pseudotyped virus from penetrating human lung cells. For people with normal heart function, clinical doses of both were pretty safe [109]. The information presented above implies that two common cardiac medicines, digoxin, and ouabain, could be used internationally as low-cost repurposing pharmaceuticals for anti-COVID-19 therapy. 8-Azaguanine is a purine analog that may have anticancer effects [110]. Although two investigations revealed 8-azaguanine as a promising therapeutic medication in COVID-19 patients [111, 112], its biological role remains unknown. Ciclopirox is a antifungal medication. According to some studies, ciclopirox has antibacterial and anti-inflammatory properties [113]. Ciclopirox may have appropriate application scenarios in COVID-19 patients with a fungal infection [114, 115]. Etoposide was another medication we discovered based on common DEGs. Etoposide is a chemotherapeutic medication that is used to treat a variety of cancers, as well as hemophagocytic lymphohistiocytosis (HLH) [116]. The numerous pathologies, clinical signs, and laboratory findings of moderate-to-severe COVID-19 were similar to those of HLH [117]. As a result, an etoposide-based medication regimen was recommended to minimize cytokine storm damage in COVID-19 patients [118, 119]. Menadione, cephaeline, cycloheximide, and pyrvinium identified in this study may be potential therapeutic agents for COVID-19. Several investigations had confirmed the efficacy of the above drugs for the treatment of COVID-19 [120, 121].

Zinc is another drug of concern [122]. Zinc could boost innate and adaptive immunity during viral infection [123]. Similarly, employing chloroquine as an ionophore could improve the efficiency of Zn [124], and Zn in infected cells could suppress SARS-COV replication [125]. As a result, the medications listed above may be used to treat COVID-19.

It is essential to highlight that the conclusions, including hub genes, regulatory networks, and medication candidates, are based on systems biology analysis. Experiments are required to confirm the biological role of the hub gene, as well as the treatment candidate's safety and efficacy.

## Conclusion

In this study, we identified common DEGs and elucidated the common molecular basis of COVID-19 and NAFLD. We constructed a PPI network using 62 common DEGs and successfully extracted the top 10 hub genes. We obtained several drug molecules associated with COVID-19 and NAFLD from the DSigDB database, providing good therapeutic recommendations. The continued prevalence of COVID-19 seems inevitable at present. In the post-epidemic era, how to provide better medical advice for people with chronic diseases is a problem we must face. Therefore, there is an urgent need for effective drugs to address the challenges posed by COVID-19. Our study identifies 10 hub genes associated with COVID-19 and NAFLD and provides new targets for drug development.

## Supplementary Information

**Additional file 1: Fig S1. **Validation of the hub genes by ROC analysis. (A) ROC analysis of the SS cohort (GSE89632); (B) was ROC analysis of the NASH cohort (GSE89632); (C) was ROC analysis of the COVID-19 cohort (GSE147507). ROC, receiver operating characteristic; SS, simple steatosis; NASH, nonalcoholic steatohepatitis; COVID-19, coronavirus disease-2019.

**Additional file 2: Table S1.** Differentially expressed genes for COVID-19.

**Additional file 3: Table S2.** Differentially expressed genes for simple steatosis.

**Additional file 4: Table S3.** Differentially expressed genes for NASH.

**Additional file 5: Table S4.** Common differentially expressed genes of SS, NASH, and COVID-19 (n = 62).

**Additional file 6: Table S5.** Gene ontology analysis.

**Additional file 7: Table S6.** Pathway enrichment analysis.

**Additional file 8: Table S7.** PPI network parameters.

**Additional file 9: Table S8.** TFs-DEGs interaction network parameters.

**Additional file 10: Table S9.** Parameters of miRNAs-DEGs interaction network.

**Additional file 11: Table S10.** Parameters of the disease-DEGs network.

## Data Availability

All data can be obtained from the GEO database website.
