# Supplementary figures and images for: Systems biology approach reveals a common molecular basis for COVID-19 and non-alcoholic fatty liver disease (NAFLD)
Source: Eur J Med Res. 2022 Nov 15;27:251. doi: 10.1186/s40001-022-00865-y (PMC9664052; doi:10.1186/s40001-022-00865-y)

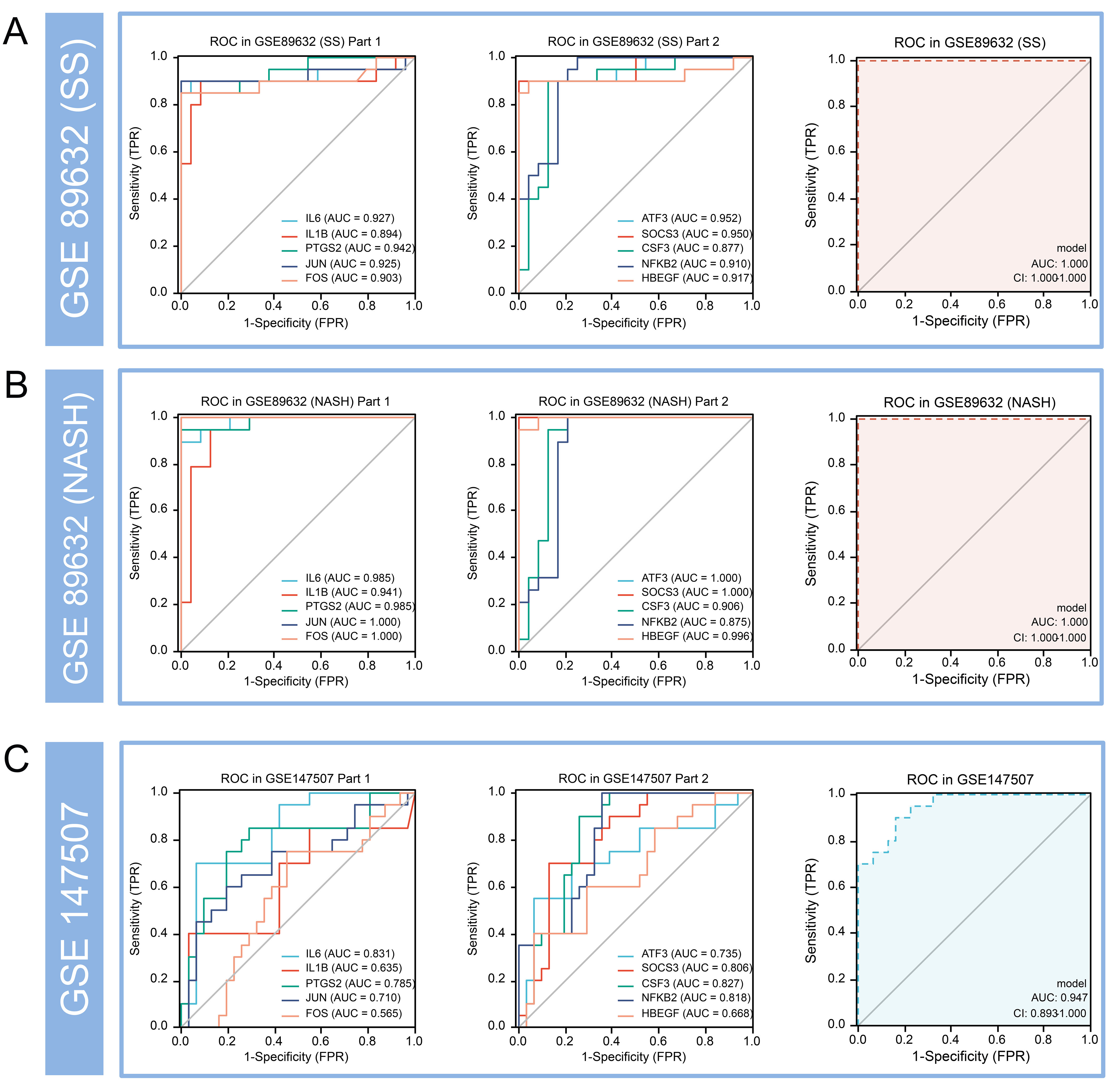

Supplement: Supplementary file 1 — Additional file 1: Fig S1. Validation of the hub genes by ROC analysis. (A) ROC analysis of the SS cohort (GSE89632); (B) was ROC analysis of the NASH cohort (GSE89632); (C) was ROC analysis of the COVID-19 cohort (GSE147507). ROC, receiver operating characteristic; SS, simple steatosis; NASH, nonalcoholic steatohepatitis; COVID-19, coronavirus disease-2019. [file 40001_2022_865_MOESM1_ESM.jpg]
